# A load-based mechanism for inter-leg coordination in insects

**DOI:** 10.1098/rspb.2017.1755

**Published:** 2017-11-29

**Authors:** Chris J. Dallmann, Thierry Hoinville, Volker Dürr, Josef Schmitz

**Affiliations:** 1 Department of Biological Cybernetics, Faculty of Biology, Bielefeld University, Bielefeld, 33615, Germany; 2 Cognitive Interaction Technology Center of Excellence, Bielefeld University, Bielefeld, 33615, Germany

**Keywords:** motor control, insect locomotion, stance-to-swing transition, campaniform sensilla, ground reaction force, electromyography

## Abstract

Animals rely on an adaptive coordination of legs during walking. However, which specific mechanisms underlie coordination during natural locomotion remains largely unknown. One hypothesis is that legs can be coordinated mechanically based on a transfer of body load from one leg to another. To test this hypothesis, we simultaneously recorded leg kinematics, ground reaction forces and muscle activity in freely walking stick insects (*Carausius morosus*). Based on torque calculations, we show that load sensors (campaniform sensilla) at the proximal leg joints are well suited to encode the unloading of the leg in individual steps. The unloading coincides with a switch from stance to swing muscle activity, consistent with a load reflex promoting the stance-to-swing transition. Moreover, a mechanical simulation reveals that the unloading can be ascribed to the loading of a specific neighbouring leg, making it exploitable for inter-leg coordination. We propose that mechanically mediated load-based coordination is used across insects analogously to mammals.

## 1. Introduction

Adaptive coordination of multiple legs is key for walking animals and robots. It ensures stability and propulsion of the body despite changes in locomotion speed or the environment. Inter-leg coordination is generally thought to arise from interconnected neural networks in the spinal cord or ventral nerve cord, which are regulated by descending inputs from the brain and afferent inputs from the legs [1–3]. However, the specific mechanisms at work during natural locomotion—when the body mechanically interacts with the environment—remain largely unknown.

One hypothesis is that mechanical interactions directly contribute to adaptive coordination [4]. In the case of walking, for example, body load is transferred among legs that are mechanically coupled through the ground. In theory, a leg in stance could start its stance-to-swing transition once its load sensors detect an unloading induced by the touch-down and subsequent loading of a neighbouring leg. That is, an intra-leg load reflex could couple the leg's step cycle to that of the neighbouring leg, simply by exploiting the load transfer between legs. Such a decentralized, load-based coordination mechanism would be a fast, computationally inexpensive and inherently adaptive complement to neural coordination signals between legs.

Recent experiments on a minimalistic four-legged robot suggest that the mechanical coupling of legs can indeed be exploited for inter-leg coordination [5,6]. In these experiments, local load feedback from the legs was sufficient to couple otherwise uncoupled leg oscillators and generate different stepping patterns. As mechanical coupling of legs is a hallmark of walking, this could also be a common control strategy in animals. In mammals, an intra-leg load reflex could be mediated in part by load-sensitive Golgi tendon organs (GTOs) [7,8]. While a leg is under load, feedback from GTOs is thought to sustain the stance phase by exciting extensor (stance) and inhibiting flexor (swing) motor neurons. Once the leg is being unloaded, feedback from GTOs decreases (GTOs do not encode load decreases). Simulation studies suggest that this can facilitate the stance-to-swing transition and might suffice to coordinate two contralateral legs [9,10]. In insects, an intra-leg load reflex could be mediated by load-sensitive campaniform sensilla. These mechanosensors detect load as strain in the cuticle [11,12]. Analogously to GTOs, campaniform sensilla have been implicated in facilitating the stance-to-swing transition once the leg is being unloaded [13–15]. Moreover, activity of campaniform sensilla on the tibia was found to coincide with the touch-down of a neighbouring leg, suggesting they signal load transfer between legs [15].

However, it has been difficult to show that the mechanical coupling of legs can be decoded and exploited for inter-leg coordination in natural locomotion. One reason is that recordings of load sensors in freely moving animals have been limited to a few accessible subgroups (for example, campaniform sensilla on the insect tibia [15–17]). Another reason is that changes in leg motor activity could not be measured simultaneously with changes in leg load; this, however, would be required to study intra-leg load reflexes during walking. Finally, the mechanical coupling of legs has not been quantified. This is critical, because load must be transferred effectively between specific legs to be exploitable for coordination. Such specific load transfer might be obvious in a biped, but cannot be intuitively inferred if more than two legs are mechanically coupled.

**Figure 1.**
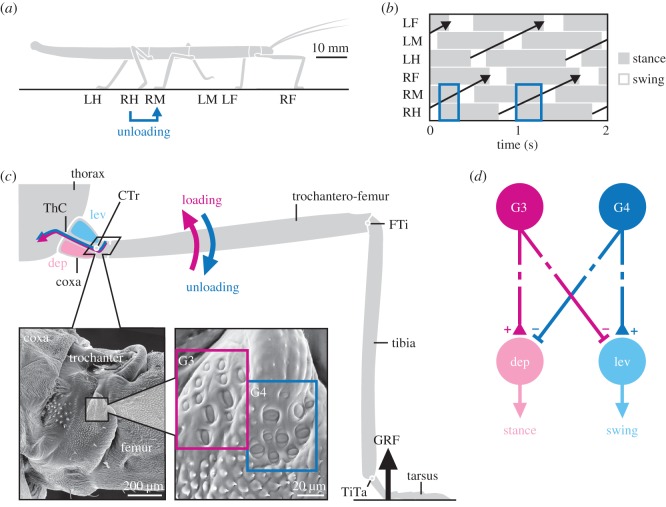
Load-based inter-leg coordination in an insect. (*a*) Schematic side view of a stick insect during walking. Each leg is labelled as being a right (R) or left (L) and a front (F), middle (M) or hind (H) leg. If a leg touches down (e.g. RH), it may unload the leg in front (e.g. RM). (*b*) Example coordination pattern of stance (grey) and swing (white) phases for each leg. Legs are coordinated in a back-to-front sequence (black arrows). Stance phases of ipsilateral neighbouring legs overlap, providing potential time for load transfer (e.g. RM and RH). (*c*) Schematic front view of a stick insect leg during stance. The GRF induces high bending torques at the proximal CTr joint. Campaniform sensilla groups G3 and G4 on the dorsal trochanter are highly sensitive to the associated strain in the trochantero-femur. G3 is activated when dorsal bending torques increase (loading of leg), G4 when they decrease (unloading of leg). (*d*) Schematic of G3/G4 reflex pathways onto coxal muscles in active animals. Broken lines indicate functional motor effects. G3 afferent activity excites (+) the depressor (stance) muscle and inhibits (−) the levator (swing) muscle [18]. G4 afferent activity is assumed to have the opposite effect. Unloading induced by a neighbouring leg may reverse afferent activity from G3 to G4, thereby promoting the leg's stance-to-swing transition. (Online version in colour.)

Here, we address these issues in freely walking stick insects (*Carausius morosus*, figure 1*a*), using a combination of motion capture, ground reaction force (GRF) measurements, electromyography and modelling. Stick insects are important invertebrate model systems for mechanosensation and motor control [1,19–21]. Like other insects, they move their legs in a back-to-front sequence during walking (figure 1*b*). Importantly, their primary load sensors, campaniform sensilla, are comparatively well described, from physiology to motor effects [18,22–25]. Moreover, the large size and sprawled posture of stick insect legs permits measuring joint torques and thus loading/unloading of individual leg segments during unrestrained walking [26]. Here, we use this unique advantage to relate the known encoding properties of campaniform sensilla directly to behaviour.

We find that campaniform sensilla at the proximal leg joints are well suited to encode the unloading of the leg during stance. The onset of unloading is strongly correlated with a change from stance-to-swing muscle activity, in agreement with an intra-leg load reflex promoting the stance-to-swing transition. Moreover, a mechanical simulation reveals that the unloading of a leg can be specifically ascribed to the loading of the ipsilateral posterior leg. This indicates that the mechanical coupling of legs during walking can directly contribute to establish adaptive inter-leg coordination.

## 2. Material and methods

We used 20 adult, female *C. morosus* reared in a laboratory colony (body mass: 0.9 ± 0.1 g, mean ± s.d.). Animals walked along a horizontal walkway (40 × 500 mm). A subset of 12 animals was used to record leg kinematics and dynamics (electronic supplementary material, video S1). These are the same animals elaborated in [26]. A subset of eight animals was used to additionally record muscle activities (electronic supplementary material, video S2).

### (a) Leg kinematics and dynamics

Kinematics and dynamics were determined as described previously [26]. In brief, kinematics were calculated from lightweight (4 mg) motion capture markers attached to the insect's body and leg segments. We used either 17 markers to capture movements of all legs or eight markers to capture movements of a subset of legs. Markers were tracked with a Vicon system at 200 Hz (Vicon MX10 with eight T10 cameras, controlled by software Nexus 1.8.5; Vicon, Oxford, UK). For visual validation, we used an additional digital video camera (Basler A602fc, Ahrensburg, Germany) recording a synchronized side view (figure 3*a*). GRFs were recorded from individual right legs at 1 kHz using three-dimensional, strain-gauge-based force plates integrated in the walkway (figure 3*a*). Kinematic and GRF data were low-pass filtered with a zero-lag, fourth-order Butterworth filter using cut-off frequencies of 20 and 10 Hz, respectively. Torques at the leg joints were derived from inverse dynamics calculations in Matlab (The MathWorks, Natick, MA, USA), which combined the single-leg kinematic and GRF data in a rigid body model of the leg. The coxa-trochanter (CTr) joint was modelled as a hinge with one degree of freedom. As described previously [26], we considered it to be directly connected to the thorax. We thereby overestimated the lever arm of the joint by approximately the length of the coxa (approx. 1.5 mm). This resulted in slightly larger torque magnitudes [26], but did not affect the conclusions reached in this study. Note that we calculated *net* torques, which represent the combined action of all forces acting in the plane of joint movement and can thus be directly related to campaniform sensilla activation.

### (b) Muscle activity

We recorded electromyograms (EMGs) from the coxal parts of the levator and depressor trochanteris muscles of right middle legs simultaneously with joint kinematics and GRFs. EMGs of each muscle were recorded with a pair of copper wires (35 µm diameter, insulated except for the tips). Wires were implanted through small holes in the cuticle made with an insect pin and held in place with dental glue. Correct electrode implantation was verified using standard criteria including resistance reflex responses to imposed movements of the CTr joint. We designed a lightweight EMG backpack (50 mg aluminium hook, figure 3*a*) to direct the electrodes to the amplifiers without risking entanglement with the legs during walking. The backpack was fixated with bees wax close to the centre of mass (COM) just behind the hind leg coxae so as to affect overall body dynamics only minimally. The backpack was connected to a string fixed midway above the walkway. String and electrodes were twisted to form a loose tether, allowing for unrestrained walking. Backpack attachment and electrode implantation did not affect joint kinematics (electronic supplementary material figure S3*a*).

EMG signals were amplified 5000-fold and filtered (50 Hz notch, 250 Hz high-pass, 7.5 kHz low-pass) using a custom-built amplifier (MA102, Electronics Workshop, Zoological Institute, Cologne). Filtered signals were A/D converted (Power 1401 mk II) and recorded with Spike2 with a sampling rate of 25 kHz (both Cambridge Electronic Design, Cambridge, UK). EMG recordings were synchronized with motion and GRF recordings of the Vicon system via a custom-built external trigger box. Muscle spikes were detected in Matlab based on amplitude thresholds. The levator EMG contained activity of slow and fast motor neurons (figure 3*b* and electronic supplementary material, figure S3*b*, 9–11 excitatory motor neurons innervate the muscle [27]). The depressor EMG contained activity of the fast depressor trochanteris motor neuron [27,28].

### (c) Mechanical simulation

To determine the mechanical load transfer among legs in stance, we simulated the animal as a rigid body in static equilibrium (Open Dynamics Engine v. 0.11.1). The body was idealized as a point mass. Each leg in stance was modelled as a frictionless, spherical (three degrees of freedom) joint attaching the body to the ground. For a given point in time, the simulation converged quickly to a stable GRF distribution among the legs in stance based on their positions relative to the COM and the animal's weight. The simulation was run for all stance phases of a reference leg (right middle leg in figure 4*b*, right front and hind leg in electronic supplementary material, figure S2). The CTr torque of the reference leg was obtained by combining its simulated vertical GRF with its measured joint kinematics. Given the frame-by-frame simulation in static equilibrium, the torque time course of the reference leg showed step-like changes that were directly related to the touch-down and lift-off events of the other legs (figure 4*b*).

## 3. Results

### (a) Proximal campaniform sensilla can encode the unloading of the leg during walking

To study load-based inter-leg coordination, we first asked which load sensors would be suited to reliably encode the unloading of the leg during the stance phase. We recently showed that load changes in the legs of walking stick insects are most pronounced and least variable at the proximal CTr joint [26] (figure 1*c*). This joint is primarily responsible for moving the leg up and down. During the stance phase, the main muscles controlling the joint (levator and depressor trochanteris) hold the fused trochantero-femur segment almost horizontally. As a consequence, GRFs induce particularly high bending torques at the CTr joint [26], and compressive strain on the dorsal trochantero-femur. Two groups of campaniform sensilla on the trochanter, G3 and G4 (figure 1*c*, SEM images), are highly sensitive to the magnitude and rate of dorsal bending [18]. The elliptic sensilla are preferentially excited by compression along their short axes. Owing to the mutually perpendicular orientations of G3 and G4 sensilla, an increase in dorsal bending activates G3 afferents without activating G4 afferents. Conversely, the release from dorsal bending activates G4 afferents without activating G3 afferents. In active animals, G3 afferents are known to excite the depressor (stance) muscle and inhibit the levator (swing) muscle of the leg [18] (figure 1*d*). The motor effects of G4 afferents have not been tested specifically in active animals. However, we assume that G4 afferents have antagonistic motor effects, much like it is known for mutually perpendicular groups of campaniform sensilla at other leg joints [22,23,29,30]. G3/G4 have been characterized in stick insect middle legs [18]. Therefore, our study focuses on middle legs, too (but see electronic supplementary material, figures S1 and S2 for other legs).

**Figure 2.**
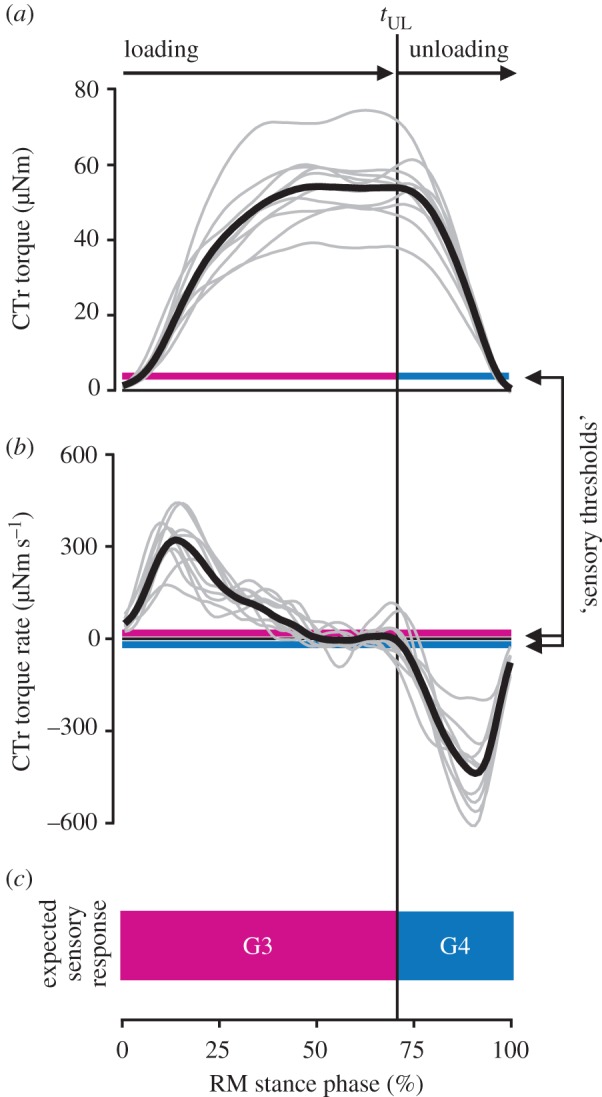
Joint torques indicate that campaniform sensilla on the trochanter encode the unloading of the leg during walking. (*a*,*b*) Torque and torque rate at the CTr joint of the right middle (RM) leg during stance. Bold black lines show the grand mean of all steps (*n* = 244 steps from *N* = 10 animals). Grey lines show means per animal. Magenta and blue lines indicate torques and torque rates above which G3/G4 afferent activity was confirmed in reduced leg preparations (‘sensory thresholds’, calculated from [18]). The vertical black line marks the mean onset of unloading, *t*_UL_. (*c*) Schematic of expected G3/G4 response based on the mean torque and torque rate time courses. Leg unloading is expected to terminate G3 and initiate G4 afferent activity. (Online version in colour.)

To test whether G3/G4 are suited to encode the unloading of the leg during walking, we analysed the time courses of torques and torque rates at the middle leg CTr joint. Torques were determined via inverse dynamics calculations based on simultaneous recordings of leg kinematics and single-leg GRFs (see Material and methods and electronic supplementary material, video S1). Upon leg touch-down, the CTr torque increased rapidly to a plateau of 54 ± 9 µNm (mean ± s.d.; *n* = 244 steps from *N* = 10 animals, figure 2*a*, loading phase). After about 70% of the stance phase, the torque decreased rapidly before the leg lifted off (unloading phase). The onset of unloading, *t*_UL_, was marked by a sudden change in torque rate peaking at −440 ± 192 µNm s^−1^ (figure 2*b*). A direct, un-masked recording of G3/G4 afferent responses to the unloading of the leg is not feasible in freely walking animals, as the afferents of both groups run in the main leg nerve (nervus cruris) together with multiple other afferent and efferent axons [27]. Therefore, we estimated the joint torques applied in previous physiological experiments on leg preparations that were effectively denervated except for G3/G4 [18]. We did so by multiplying the reported bending forces with the lever between force application and the CTr joint (an average trochanter–femur length, 11.7 mm in our experiments). The lowest torque and torque rate at which G3/G4 afferent activity was confirmed were 3.4 µNm and 10.5 µNm s^−1^, respectively (figure 2*a,b*, magenta and blue lines). These ‘sensory thresholds' lie well below the sensitivities required for reliably signalling *t*_UL_ as measured during walking. This was also the case in the front and hind legs (electronic supplementary material, figure S1). Given the directional sensitivities of G3/G4, we can thus predict that *t*_UL_ is encoded by a change from G3 to G4 afferent activity (figure 2*c*).

### (b) The onset of unloading coincides with a change from stance to swing muscle activity

Assuming the motor effects illustrated in figure 1*d*, a change from G3 to G4 afferent activity should promote the leg's stance-to-swing transition. Until *t*_UL_, the depressor muscle should be excited and the levator muscle inhibited. After *t*_UL_, the depressor muscle should be inhibited and the levator muscle excited.

**Figure 3.**
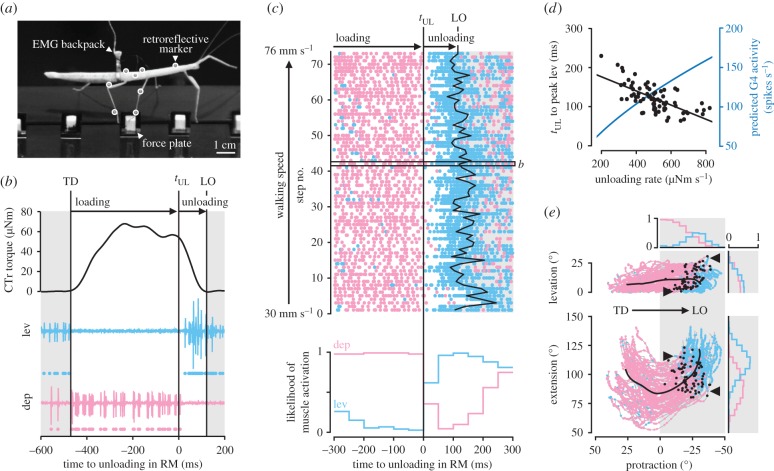
Leg unloading coincides with a switch from stance to swing muscle activity. (*a*) Side view of an animal carrying a lightweight EMG backpack and motion capture markers (white circles) while stepping onto a force plate. (*b*) CTr torque of the right middle (RM) leg and simultaneously recorded activity of the levator muscle (light blue) and depressor muscle (light red) of an example step. Dots below EMG traces indicate muscle spikes detected based on amplitude. TD, touch-down; LO, lift-off. (*c*) Raster plot of detected muscle spikes (top) and likelihood of muscle activation (bottom) relative to *t*_UL_ of the right middle leg (*n* = 73 steps from *N* = 8 animals). Walking speed corresponds to the mean speed of the COM during stance. The box marks the step shown in *b*. (*d*) Time from *t*_UL_ to peak levator activity over unloading rate (*r* = −0.67, *p* < 0.001, *n* = 73 steps from *N* = 8 animals). Peak levator activity was determined from rectified and smoothed EMG signals (moving average filter with 80-ms window). The unloading rate corresponds to the mean CTr torque rate from *t*_UL_ to LO in each step. The blue line shows the corresponding predicted G4 afferent activity (calculated from [18]). (*e*) Leg movements during the stance phases in *c* plotted in joint angle space. Coloured dots indicate detected muscle spikes. Movement direction is from left to right (positive to negative protraction). Bold black lines show the mean movement trajectory. Black dots mark *t*_UL_ in individual stance phases. Histograms indicate the likelihood of muscle activation per 5° joint angle bins in the second half of stance (grey shaded area). Arrowheads mark the variable onset of the levator in two exemplary stance phases. At zero degree protraction and levation, the leg is perpendicular to the long body axis in the horizontal plane; at zero degree extension, the tibia is completely flexed. (Online version in colour.)

To test whether *t*_UL_ is in fact reliably followed by a change in muscle activity, we recorded EMGs from the levator and depressor muscles of right middle legs simultaneously with leg kinematics and GRFs (see Materials and methods and electronic supplementary material, video S2). Animals carried a lightweight EMG backpack (figure 3*a*), from which electrodes were implanted in the coxa of the middle leg. To correlate muscle activity with *t*_UL_, we first determined *t*_UL_ in each stance phase. This was possible because the variability of torque time courses is particularly low at the CTr joint [26]. Specifically, we used the time point at which the CTr torque rate reached 25% of the maximum unloading rate during the stance phase. This relative, non-zero threshold allowed us to reliably detect the rapid decrease in torque toward the end of stance in individual steps with negligible delay, while ignoring small fluctuations around the plateau earlier in stance (see example in figure 3*b*).

Figure 3*b* shows that levator activity started only after *t*_UL_ and before the leg was lifted off the ground. Conversely, depressor activity was high during the loading phase and terminated with *t*_UL_. A similar pattern of motor activity was present in all steps recorded, independent of walking speed (*n* = 73 steps from *N* = 8 animals, figure 3*c*, top, see also electronic supplementary material, figure S3*b*). To quantify the change in muscle activity, we determined the likelihood of muscle activation relative to *t*_UL_ by checking for the presence of a motor spike in every 50-ms time bin before and after the event. In agreement with the predicted motor effects of G3/G4 afferents, the likelihood of depressor activity declined abruptly after *t*_UL_, whereas the likelihood of levator activity strongly increased (figure 3*c*, bottom). Note that depressor activity was much stronger than in tethered animals walking with body load supported [31,32]. This would be expected if depressor activity were driven in part by excitatory feedback from G3, and suggests that the depressor muscle contributes to body support and propulsion in free walking [26,33,34]—a somewhat neglected aspect in current control models [35,36]. Levator activity after *t*_UL_ could result from decreased inhibitory feedback from G3. Note here that levator activity and, consequently, the lift-off of the leg were variable with respect to *t*_UL_ (figure 3*c*, top). Part of this variability can be explained by step-to-step variations in unloading rate: the higher the unloading rate, the earlier the peak levator activity (figure 3*d*, *r* = −0.67, *p* < 0.001). This would be expected if levator activity were driven in part by excitatory feedback from G4, because G4 afferent activity increases with increasing unloading rate [18] (figure 3*d*, blue line).

In principle, levator activity could also be initiated by position and movement signals from the leg. For example, flexion/extension of the tibia is signalled by the femoral chordotonal organ [37,38], and leg levation is signalled by a hair plate on the trochanter [28] and an internal strand receptor [39]. However, the onset of levator activity did not correlate strongly with any specific joint angle or change in movement direction, such as from flexion to extension (figure 3*e*). Rather, the levator started firing at various leg angles from step to step (figure 3*e*, arrowheads). Accordingly, the likelihood of muscle activity did not change abruptly with any one postural parameter (figure 3*e*, histograms), contrary to the observed change with *t*_UL_. The latter did not correlate strongly with any specific joint angle either (figure 3*e*, black dots).

Taken together, these results indicate that load feedback from G3/G4 afferents can reliably promote the leg's stance-to-swing transition on a step-by-step basis.

### (c) Unloading of a leg can be specifically ascribed to loading of the ipsilateral posterior leg

The patterns of coxal muscle activity indicate that *t*_UL_ is not induced by the leg itself. Levator activity started only after *t*_UL_, and given the slow temporal filter properties of insect muscle [40], relaxation of the depressor must be expected to occur only after *t*_UL_ as well. Therefore, we tested whether *t*_UL_ and the ensuing change in muscle activity could be ascribed to mechanical load transfer within a specific pair of legs. This would be required for proper inter-leg coordination to emerge. If so, one would predict that *t*_UL_ is closely preceded by the touch-down of another leg, and that this leg can significantly unload the middle leg on a step-by-step basis.

**Figure 4.**
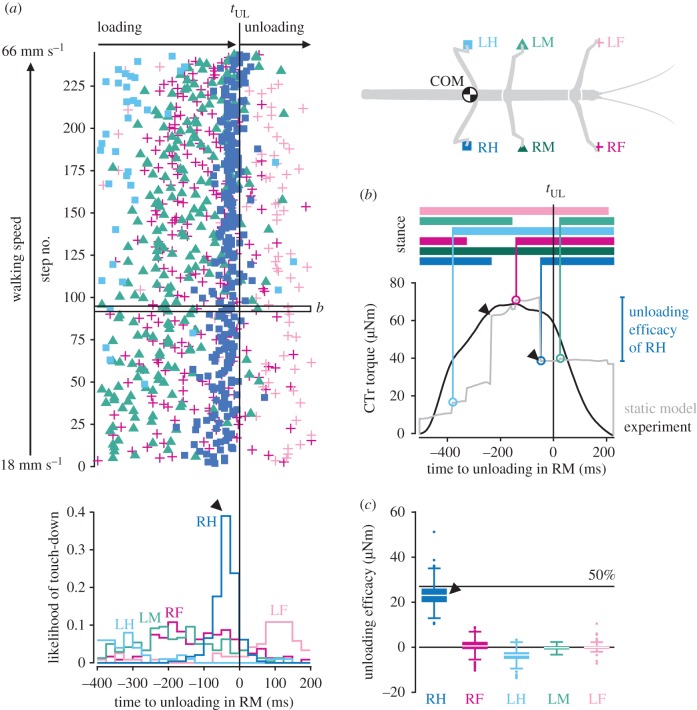
Leg unloading is linked to loading of posterior neighbouring leg. (*a*) Touch-down events of all legs (top) and likelihood of touch-down per leg (bottom) relative to the onset of unloading, *t*_UL_, in the right middle (RM) leg (*n* = 244 steps from *N* = 10 animals). Touch-downs of the right hind (RH) leg reliably precede *t*_UL_ in the middle leg with short latency (arrowhead). The box marks the step shown in *b*. (*b*) Exemplary stance phase showing the CTr torque of the right middle leg from the experiment (black) and the static model (grey). Horizontal bars on top indicate the stance phases of all legs. Vertical lines highlight the times of leg touch-downs. Touch-down induced changes in simulated torque provide a measure of a leg's unloading efficacy. (*c*) Simulated efficacy of legs in unloading the right middle leg, pooled across all steps. The 50% line corresponds to 50% of the mean torque measured during walking (figure 2*a*). (Online version in colour.)

To test for temporal correlation, we determined *t*_UL_ in individual steps of the right middle leg as before. In addition, we automatically detected the stance phases of all other legs relative to it based on the velocity of their tibia–tarsus joints (electronic supplementary material figure S4*a*). Detected stance phases corresponded well with manually identified stance phases based on GRFs and digital video data (average difference less than 20 ms, electronic supplementary material figure S4*b*). Figure 4*a* shows that *t*_UL_ was indeed closely preceded by the touch-down of a leg over the entire range of walking speeds. The temporal correlation was strongest with the right hind leg. It touched down most frequently 25–50 ms before *t*_UL_ (figure 4*a*, arrowhead), or 35 ± 33 ms on average (*n* = 244 steps from *N* = 10 animals). By contrast, the relative touch-down times of the other legs were more scattered and did not coincide with middle leg unloading.

Assessing the magnitude of unloading in one leg due to loading of another is difficult in the experimentally measured torque profiles. Therefore, we used a mechanical simulation of the animal in static equilibrium (see Material and methods). The example time course depicted in figure 4*b* shows the typical step-like increases and decreases in simulated torque induced by touch-downs and lift-offs of the other legs, with the right hind leg having the largest effect (arrowheads). Conveniently, the step-like decreases in torque provide a direct measure of the maximum unloading achievable through pure mechanical coupling, which we define as ‘unloading efficacy.' Comparing the unloading efficacy across legs revealed that the right hind leg had the largest effect in all steps (figure 4*c*, arrowhead). On average, it unloaded the middle leg by 23 ± 5 µNm (*n* = 244 steps from *N* = 10 animals). This corresponds to almost 50% of the CTr torque magnitude measured during walking (figure 2*a*)—the theoretical maximum load transfer between two neighbouring legs in static equilibrium. In contrast, the unloading effects of the other legs were significantly weaker (ANOVA; *F*_4,876_ = 2069.15, *p* < 0.001; Tukey's HSD post hoc test at the 0.05 level of significance). The effects were consistent across animals.

Taken together, the timing and magnitude of load transfer suggest that the unloading of the middle leg can be caused purely mechanically by the touch-down of the ipsilateral hind leg. Load transfer was similarly effective between ipsilateral front and middle legs (electronic supplementary material figure S2).

## 4. Discussion

In this study, we tested whether animals can exploit the mechanical coupling of their legs during walking to establish adaptive inter-leg coordination. To this end, we provided the first simultaneous recordings of leg kinematics, GRFs and muscle activity in a freely walking insect and a mechanical simulation of load transfer among legs. Our results suggest that the touch-down of a leg effectively unloads the neighbouring leg in front, which can reliably detect the unloading and elicit motor effects promoting its stance-to-swing transition. This indicates that neighbouring legs can be coordinated in a back-to-front sequence during walking based on their mechanical coupling through the ground.

Our results provide new insight into the flexible leg coordination found in insects [41–44]. Intriguingly, much of this flexibility can be accounted for when assuming a simple set of coordination rules acting between direct neighbouring legs (‘Cruse rules' [21,36,45]). Two core rules state that a leg's stance-to-swing transition is suppressed while the posterior neighbouring leg is in swing (rule 1), but promoted as soon as the latter has touched down (rule 2). The load-based coordination mechanism described here could readily be a corresponding neuromechanical implementation. For example, while the hind leg is in swing, load of the middle leg is high. Local load feedback can then reinforce ongoing stance muscle activity in the middle leg and suppress the leg's stance-to-swing transition (rule 1). When the hind leg touches down, load of the middle leg effectively decreases due to mechanical coupling. The altered local load feedback can then promote the stance-to-swing transition of the middle leg (rule 2).

The hypothesis that campaniform sensilla on the trochanter mediate this feedback is supported by previous observations. For example, one study in stick insects described that continuous pressure on the trochanter can suppress the leg's stance-to-swing transition [13]. Other studies in the stick insect, locust and cockroach, in which distal parts of a leg were denervated or replaced by a prosthesis, suggest that load feedback from proximal campaniform sensilla might be sufficient for coordination [46–48]. By linking biomechanics and mechanosensation in freely walking insects, our study builds upon these previous results in several ways. First, joint torque calculations allowed us to predict that G3/G4 are suited to reliably encode the unloading of the leg on a step-by-step basis. Second, relating leg motor activity directly to changes in leg load allowed us to investigate the corresponding intra-leg load reflex. Third, simulating the load transfer among legs revealed that, through mechanical coupling, the load reflex can contribute to inter-leg coordination.

In natural locomotion, coordination likely results from integration of load feedback with other somatosensory feedback from the same and neighbouring legs, as well as input from the brain [1]. For example, G3/G4 feedback could be integrated with feedback from tibial and femoral campaniform sensilla local to the leg. These sensilla too respond to load changes in the plane of leg levation/depression [23,25]. Indeed, activity of tibial campaniform sensilla has been suggested to reflect the unloading of the leg in cockroaches and locusts [14,15]. In addition, G3/G4 feedback could be integrated with feedback from position and movement sensors of the leg [49]. For example, levator and depressor muscle activity could be modulated by input from the femoral chordotonal organ, which signals the position and movement of the tibia [37,38]. We found no strong correlation between the change in muscle activity and any one postural parameter (figure 3*e*). However, it remains a possibility that a combination of changes in position and movement could contribute to timing the stance-to-swing transition [50]. Finally, G3/G4 feedback could be integrated with proprioceptive signals from other legs [51–54]. For example, campaniform sensilla on the hind leg could signal ground contact to the middle leg via polysynaptic pathways. This might explain why basic coordination patterns can be generated across legs even in the absence of mechanical coupling [32,55–57]. Nevertheless, our results strongly suggest that mechanical coupling is exploited in natural locomotion to complement neural coordination signals between legs. To further understand this interplay, future studies might benefit from combining detailed biomechanical analyses like ours with genetic techniques [19] to selectively manipulate load feedback during unrestrained behaviour.

It is plausible to assume that load-based coordination is used across insect species. Groups of campaniform sensilla similar to G3/G4 in stick insects (mutually perpendicular orientations in the plane of leg movement) are present on proximal leg segments of other insect species, including cockroaches [58], locusts [59], blowflies and *Drosophila* [60,61]. Although the load experienced by individual leg segments will depend on leg morphology, posture and body weight, cuticular strains can be expected particularly high on proximal leg segments. Therefore, these campaniform sensilla might well be similarly suited to reliably encode the mechanical interactions of legs during locomotion. Importantly, a mechanical stimulus applied to the tarsus can propagate to campaniform sensilla on the trochanter almost instantly (less than 1 ms in a locust leg [62]). This might enable rapid local reflexes [62] that mediate inter-leg coordination even at relatively high walking speeds.

The main features of the load-based coordination mechanism in stick insects also parallel findings in mammals. Studies in cats indicate that load feedback from GTOs reinforces extensor (stance) and inhibits flexor (swing) muscle activity while the leg is under load [7,8], analogously to load feedback from campaniform sensilla G3. Interestingly, simulation studies in cats and humans predict that this load feedback, rather than position feedback from the hip, leads to stable coordination between contralateral legs [9,10]. Recent experiments in freely moving mice, in which proprioceptive feedback could be eliminated genetically, confirm a dominant role of local load feedback in coordination [63].

Together, these results indicate the possibility that mechanically mediated load-based coordination is a widespread control strategy. Implemented in multi-legged robots, the mechanism described here could provide a computationally inexpensive, robust and inherently adaptive alternative to control strategies based on explicit kinematic models.

## Supplementary Material

Supplementary Figures

## Data accessibility

The data used for this study have been deposited in Dryad as http://dx.doi.org/10.5061/dryad.8d0h5 [64].
