# Supplementary material for: A load-based mechanism for inter-leg coordination in insects
Source: Proc Biol Sci. 2017 Nov 29;284(1868):20171755. doi: 10.1098/rspb.2017.1755 (PMC5740276; doi:10.1098/rspb.2017.1755)
Supplement: Supplementary Figures [file rspb20171755supp1.pdf]

Supplementary Figures

A load-based mechanism for inter-leg coordination in insects

Chris J. Dallmann[1,2], Thierry Hoinville[1,2], Volker Dürr[1,2] and Josef Schmitz[1,2]

[1]Department of Biological Cybernetics, Faculty of Biology, and [2]Cognitive Interaction Technology Center of Excellence, Bielefeld University, Bielefeld 33615, Germany

http://dx.doi.org/10.1098/rspb.2017.1755

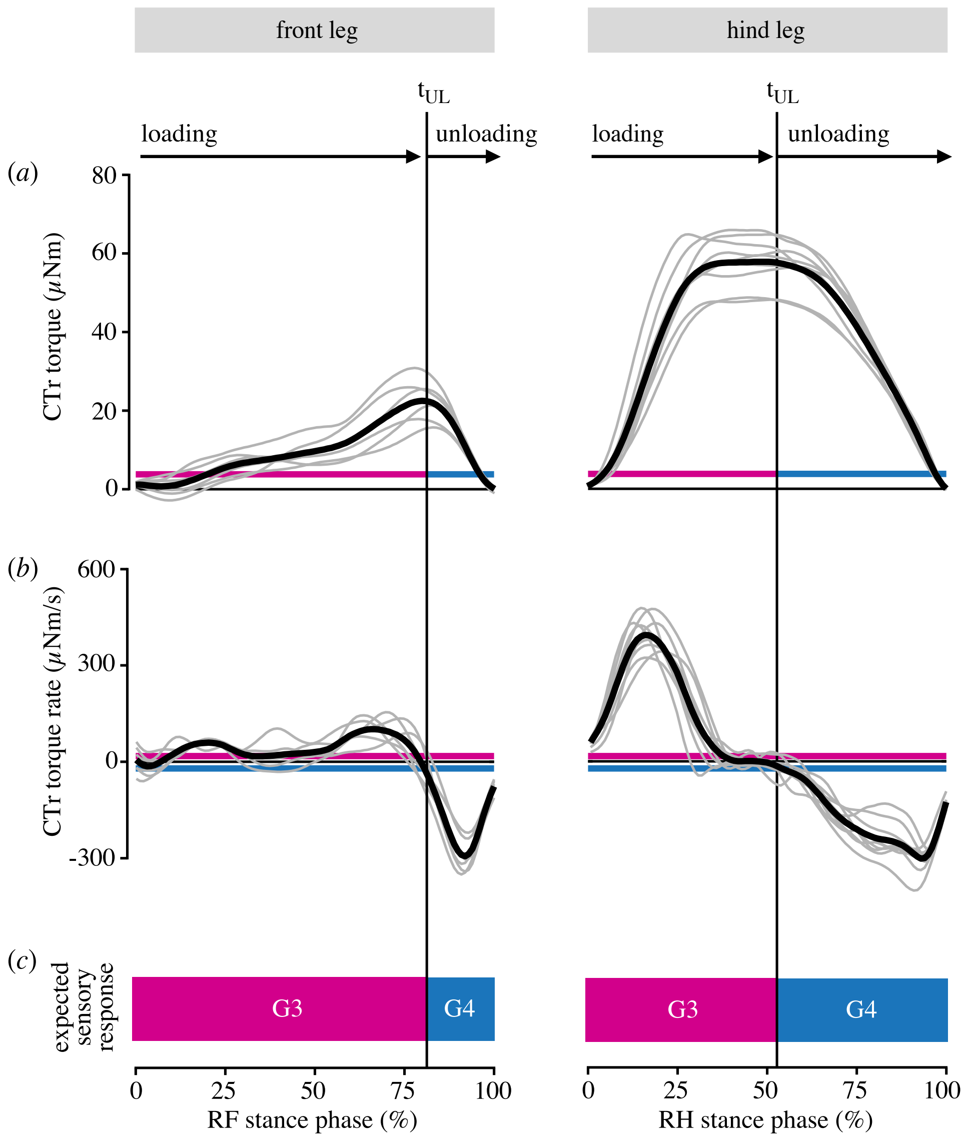

Figure S1. Joint torques of front and hind legs indicate that campaniform sensilla on the trochanter encode the unloading of the legs during walking. (*a*–*b*) Torque and torque rate at the CTr joint of the right front (RF) and right hind (RH) leg during stance. Bold black lines show the grand mean of all steps (RF, n = 140 steps from N = 6 animals; RH, n = 427 steps from N = 9 animals). Gray lines show means per animal. Magenta and blue lines indicate torques and torque rates above which G3/G4 afferent activity was confirmed in reduced leg preparations (calculated from [23]). The vertical black line marks the onset of unloading, t_{UL}. (*c*) Schematic of expected G3/G4 response in front and hind legs based on the mean torque and torque rate time courses. As in middle legs, leg unloading is expected to terminate G3 and initiate G4 afferent activity.

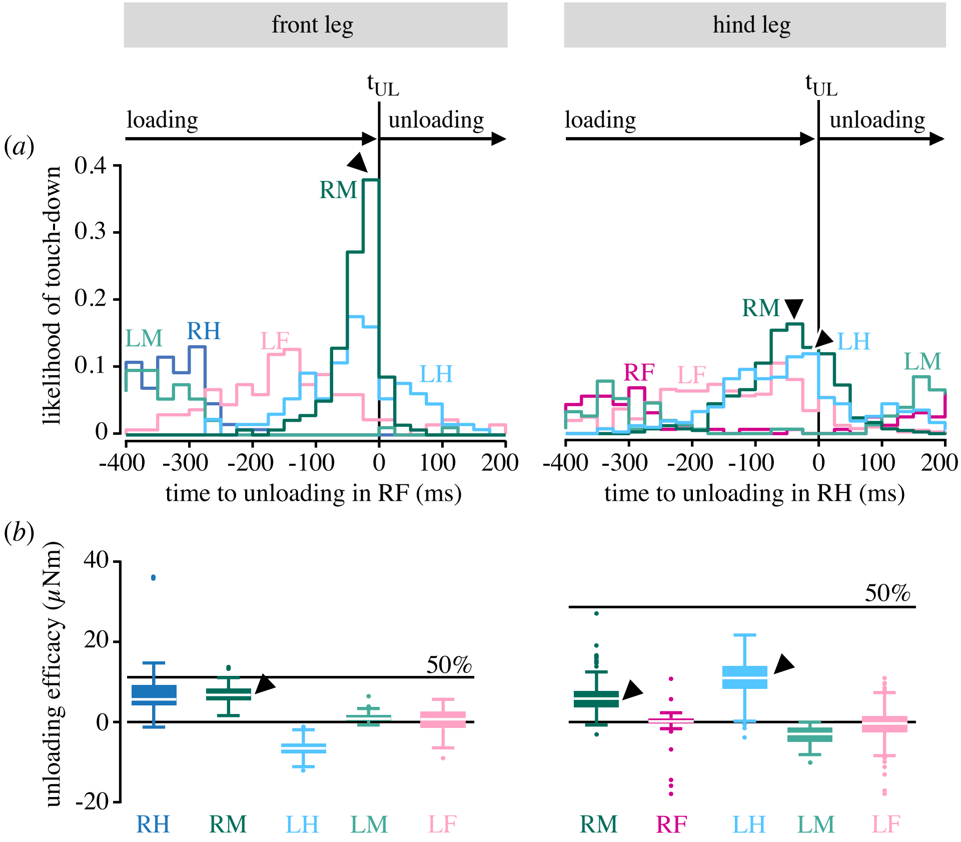

Figure S2. Unloading effects in front and hind legs due to mechanical coupling. (*a*) Likelihood of touch-down per leg relative to the onset of unloading, t_{UL}, in the right front (RF) and right hind (RH) leg (RF, n = 140 steps from N = 6 animals; RH, n = 427 steps from N = 9 animals). Unloading of the right front leg is closely preceded by the touch-down of the right middle (RM) leg (arrowhead left). Unloading of the right hind leg is preceded by touch-downs of both the right middle and the left hind (LH) leg (arrowheads right), but not any one leg alone. (*b*) Efficacy of legs in unloading the front and hind legs, pooled across steps. The 50%-lines corresponds to 50% of the mean CTr torques measured during walking (see supplementary figure S1*a*). The right front leg can be unloaded by both ipsilateral legs. However, only the touch-downs of the middle leg (arrowhead left) coincide with front leg unloading (see *a*). The right hind leg can be unloaded by both the right middle and the left hind leg (arrowheads right). Their touch-downs also coincide with hind leg unloading (see *a*). Note though that the unloading effect is considerably lower compared with the unloading effects of the hind on the middle leg (see figure 4*c*) and the middle on the front leg (see *b*). Thus, the load-based mechanism depicted in figure 1 might be particularly effective in coordinating ipsilateral legs in a back-to-front sequence.

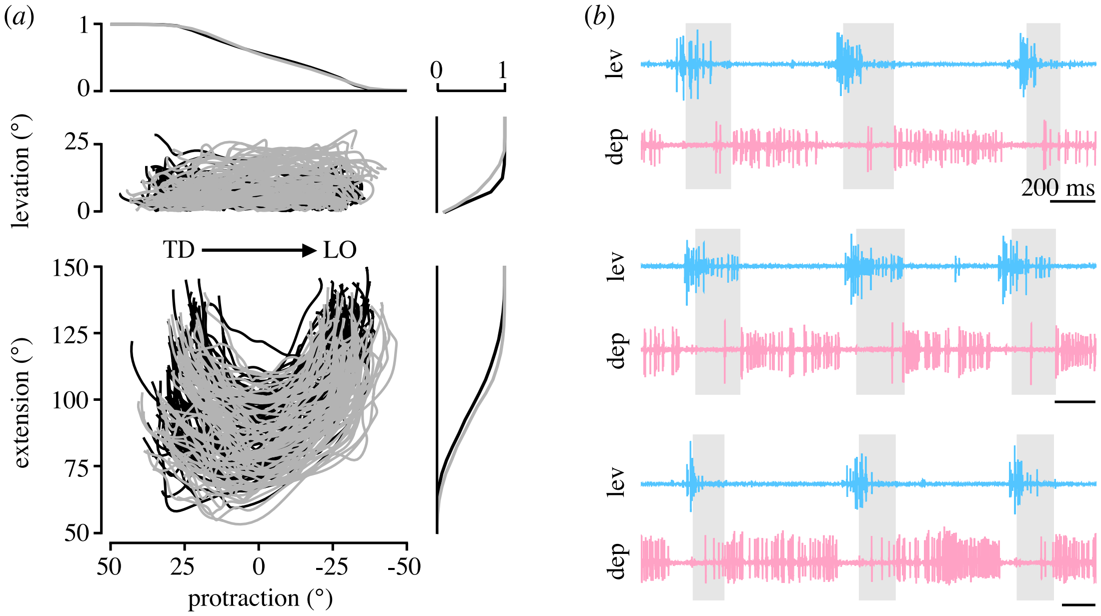

Figure S3. (*a*) Electrode implantation and backpack attachment do not affect intra-leg coordination. Levation and extension angles versus protraction angles and their cumulative probabilities in right middle legs. Gray lines show data with EMG backpack attached and electrodes implanted (n = 73 steps from N = 8 animals, see figure 3). Black lines show data recorded from the same animals prior to backpack attachment and electrode implantation (n = 80 steps from N = 8 animals). Movement direction is from left to right (positive to negative protraction). TD, touch-down, LO, lift-off. (*b*) Example EMG recordings of the coxal levator (swing) muscle and the coxal depressor (stance) muscle of right middle legs during unrestrained walking. Examples are from three different animals walking at slightly different speeds (note different lengths of scale bars, about 45 mm/s on average). Gray boxes indicate swing phases. Activity of the two muscles is largely reciprocal. Depressor activity starts during swing and is highest during stance. Main levator activity starts toward the end of stance after termination of depressor activity and continues into swing. Note that muscle activity during stance is stronger and less variable than in tethered walking animals with body load supported [31,32]. This difference could readily result from load feedback from campaniform sensilla G3/G4 (figure 1*d*). Note also that levator EMGs are multiunit recordings containing activities of slow and fast motor neurons.

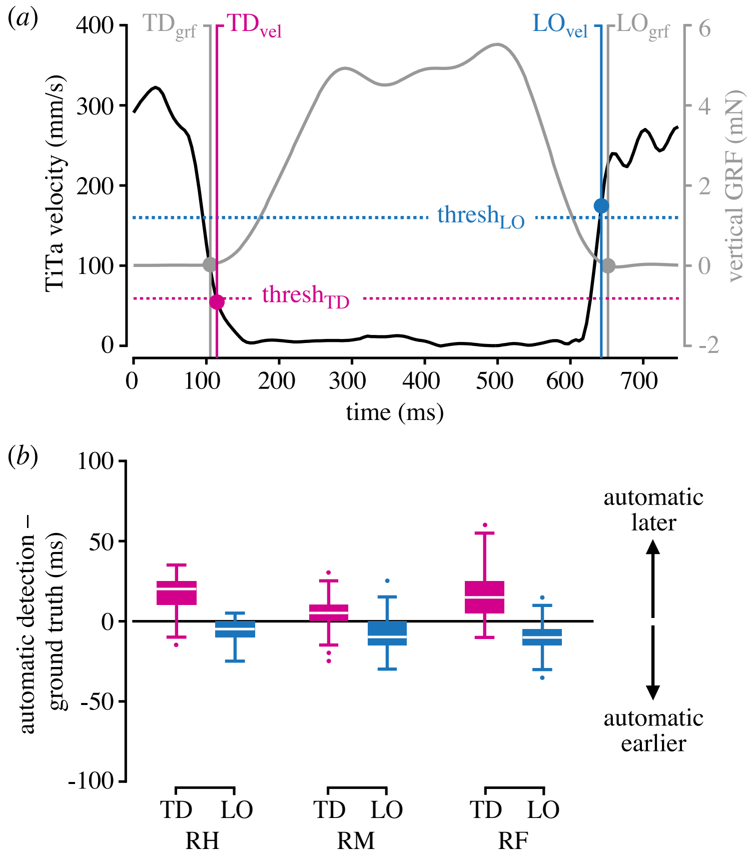

Figure S4. Automatic detection of stance phases is reliable. (*a*) Single step example of a right middle leg, illustrating the similarity of results from automatic and manual detection of leg touch-down (TD) and leg lift-off (LO). The automatic detection was based on the velocity magnitude of the tibia-tarsus joint (black) using threshold crossing (dotted lines, thresh_{TD} = 60 mm/s, thresh_{LO} = 160 mm/s). The manual detection was based on the vertical ground reaction force (GRF) of the leg (gray). The delay between automatically and manually detected events is small (10 ms, equivalent to two motion capture sampling points). (*b*) Delays between automatically and manually (ground truth) detected stance phases for all reference steps. The automatic detection worked reliably with small delays only. The sample numbers are: right hind (RH) leg, n = 427 steps from N = 9 animals; right middle (RM) leg, n = 244 steps from N = 10 animals; right front (RF) leg, n = 140 steps from N = 6 animals.
